# TNF-α mediated apoptosis plays an important role in the development of early diabetic retinopathy and long-term histopathological alterations

**Published:** 2009-07-25

**Authors:** Antonia M. Joussen, Sven Doehmen, Minh L. Le, Kan Koizumi, Sven Radetzky, Tim U. Krohne, Vassiliki Poulaki, Irina Semkova, Norbert Kociok

**Affiliations:** 1 Department of Ophthalmology, Heinrich Heine University, Duesseldorf, Germany; 2 Department of Vitreoretinal Surgery, Center for Ophthalmology, University of Cologne, Germany; 3 Center for Molecular Medicine, University of Cologne, Germany; 4 Massachussetts Eye and Ear Infirmary, Harvard Medical School, Boston, MA

## Abstract

**Purpose:**

The pathophysiology of diabetic retinopathy involves leukocyte adhesion to retinal vasculature, early blood-retinal barrier breakdown, capillary nonperfusion, and endothelial cell death. We investigated the involvement of tumor necrosis factor α (TNF-α) in diabetes-related histopathological changes in two relevant rodent models.

**Methods:**

In short-term studies, Long-Evans rats with streptozotocin–induced diabetes were treated with or without the TNF-α inhibitor, etanercept. For long-term studies, tumor necrosis factor receptor I (TNF-RI)-deficient mice and TNF-RII-deficient mice, as well as C57/Bl6 wild-type mice, were fed 30% galactose for up to 20 months. The retinal histopathological alterations of hypergalactosemia were analyzed in trypsin digest preparations. Endothelial cell injury and apoptosis in rat retinas were evaluated by propidium iodide, TUNEL, CytoDeath staining, and DNA fragmentation ELISA. Caspase 3 and 8 activity was evaluated by immunoblotting and quantitative enzymatic activity assay.

**Results:**

Etanercept suppressed caspase activation, retinal cell injury, and apoptosis in short-term diabetic rats. Pericyte and endothelial cell loss were also reduced in long-term hypergalactosemic mice. Long-term studies demonstrated that pericyte loss and endothelial cell loss were reduced in comparison to wild-type diabetic controls.

**Conclusions:**

Our study identifies an important role for TNF-α in the pathogenesis of signature diabetic retinopathy pathologies and demonstrates that etanercept can inhibit retinal cell death and long-term complication of diabetes. Taken together, our results suggest that etanercept could prove beneficial in preventing both early and late vascular diabetic complications.

## Introduction

Leukocyte adhesion to the diabetic retinal vasculature results in early blood-retinal barrier breakdown, capillary nonperfusion, endothelial cell injury, and cell death. We have previously shown that intercellular adhesion molecule-1 (ICAM-1) and the leukocyte integrin CD18 are required for these processes [1,2]. We also demonstrated that leukocyte-mediated retinal cell apoptosis is among the earliest pathological manifestations of diabetic retinopathy and results in the formation of acellular-occluded capillaries, microaneurysms, and vascular basement membrane thickening [3-6]. The diabetic eye responds to the progressive vascular occlusions with an increase of vascular permeability, leading to macula edema, or the formation of neovessels that finally proliferate into the vitreous [3,4]. Various mediators, such as vascular endothelial growth factor (VEGF) and tumor necrosis factor-α (TNF-α), contribute to the upregulation of the adhesive molecules of the endothelial cells and leukocytes. Although the role of VEGF in the development of diabetic complications in the eye is well established, the role of TNF-α is still unclear.

TNF-α is the prototypical member of a family of cytokines that also include Fas ligand (FasL), CD40 ligand (CD40L) and TNF-related apoptosis inducing ligand (TRAIL), and induce apoptosis, differentiation, cell activation, and inflammation [7]. TNF-α is found in the extracellular matrix, endothelium, and vessel walls of fibrovascular tissue of proliferative diabetic retinopathy (PDR) [8] and is elevated in the vitreous from eyes with this complication [9,10]. The susceptibility to diabetic retinopathy has been associated with TNF-α gene polymorphism [11] and expression of human leucocyte antigen (HLA)-DR3 and HLA-DR4 phenotypes and HLA-DR4 phenotypes. The involvement of TNF-α in the pathogenesis of diabetic retinopathy [8,12,13] could be attributed, in part, to its proinflammatory activity. Indeed, we have demonstrated that TNF-α is elevated in the diabetic retina and that the soluble p75-Fc fusion protein (etanercept) suppresses leukocyte adhesion in diabetic retinal arterioles, venules, and capillaries, as well as blood-retinal barrier breakdown in a rat model of diabetic retinopathy [14].

TNF-α is a potent inducer of endothelial cell apoptosis [15,16]. Yet, its role in regulating endothelial cell apoptosis in diabetic retinopathy has not been studied. Our previous results have demonstrated a mechanism of leukocyte-mediated endothelial cell death depending on the TNF-related ligand FasL [17]. We now show that the soluble TNF-α inhibitor etanercept significantly reduces retinal cell apoptosis, caspase activation and long-term complications during the course of diabetes in the eye. Our data identify TNF-α as a key molecule in the pathogenesis of the early signature pathologies and later diabetic complications that characterize diabetic retinopathy.

## Methods

### Animals

Male Long-Evans rats purchased from Jackson Labs (Bar Harbor, ME) weighing approximately 200 g at an age of 6 weeks were used. The animals were fed standard laboratory chow and allowed free access to water in an air-conditioned room with a 12h:12h light-dark cycle. Unless otherwise stated, the animals were anesthetized with 40 mg/kg ketamine (Ketalar; Parke-Davis, Morris Plains NJ) and 4 mg/kg xylazine (Rompun, Bayer Leverkusen, Germany) before all experiments. For long-term experiments, mice deficient for TNF-α receptor protein 55 (Rp55; Rp55^−/−^, B6.129-Tnfrsf1a^tm1Mak^/J), and Rp75 (Rp75^−/−^, B6.129S2-Tnfrsf1b^tm1Mwm^/J) were used (Jackson Laboratory, Bar Harbor, ME). Age-matched C57BL/6J mice served as controls. Rp55^−/−^ and Rp75^−/−^ mice possess normal retinas and are markedly hypomorphic rather than null alleles for tumor necrosis factor receptor I (TNF-RI)-deficient mice and TNF-RII. Animal care guidelines comparable to those published by the Institute for Laboratory Animal Research (Guide for the Care and Use of Laboratory Animals) were followed and were approved by the Animal Care and Use Committee of the Regierungspräsidium, Köln, Germany.

### Rat model of streptozotocin-induced diabetes

Animals were allowed to undergo a 12 h fast, following which they received a single 60 mg/kg intraperitoneal injection of streptozotocin (STZ; Sigma, St. Louis, MO) in 10 mM sodium citrate buffer, pH 4.5 or citrate buffer alone. Then 24 h later, animals with blood glucose levels higher than 250 mg/dl were considered diabetic. In vivo experiments were performed two weeks following STZ injection, and blood glucose was measured again before the onset of the experiments. In previous studies, we had demonstrated that STZ nonconverters do not differ in term of gene expression, amount of blood-retinal barrier breakdown, or leukostasis from nondiabetic controls [18]. Thus, in the current study nondiabetic animals with confirmed glucose levels of less than 120 mg/dl were used as controls.

### Mouse model of galactosemia

Experimental galactosemia was produced by feeding Rp55^−/−^ and Rp75^−/−^ mice and respective controls a diet of Teklad 7004 (Harlan, WI) enriched with 30% D-galactose as previously described [4,19]. This results in hyperglycemia largely devoid the attendant metabolic abnormalities associated with diabetes. Bodyweight was measured weekly. The level of the blood hexose elevation was estimated by measuring the levels of nonenzymatic hemoglobin A1c (HbA1c) using affinity chromatography (Glyc-Affin, Pierce, Rockford, Il). Mice with HbA1c of ≥6% in repeated measurements were considered galactosemic and included in the study. Using this animal model, we recently demonstrated a central role of adherent leukocytes in the pathogenesis of diabetic retinopathy [19].

### Treatment with a soluble TNF-α receptor

Soluble p75 TNF-α receptor/Fc fusion protein (Enbrel™, etanercept; Wyeth-Pharma GmbH, Münster, Germany) was reconstituted with sterile water according to the manufacturer’s instructions. Starting one day after the STZ treatment, confirmed diabetic rats were randomized to receiving either etanercept or solvent alone. The drug was administered subcutaneously. The dose was 0.3 mg/kg at three times per week according to the recommendation of the manufacturer for treatment of rheumatoid arthritis in humans and according to our previous studies in the rat model [14,20-22]. For each experiment, a minimum of n=6 animals per treatment group were used.

### Propidium iodide labeling in vivo

Dead and injured endothelial cells were labeled in vivo using propidium iodide (PI; Molecular Probes, Eugene, OR). Following the induction of deep anesthesia with 50 mg/kg intraperitoneal sodium pentobarbital, 1 mg/ml PI in PBS (140 mM NaCl, 10 mM NaH_2_PO_4_, pH 7.4) was injected intravenously via the tail vein at a concentration of 5 µmol/kg (0.668 ml/200 mg bodyweight). After fixation by intracardiac perfusion with 1% paraformaldehyde in PBS labeling with concanavalin A-lectin was performed as described before [2,19]. The identification of arterioles, venules, and capillaries was done based on morphological criteria. Venules are usually slightly larger than arterioles, and capillaries have the smallest diameter. Labeled endothelial cells were distinguished from surrounding cells, especially pericytes, by distinct cellular outline and nuclear shape of the endothelial cells on retinal flat mounts. All experiments were performed in a masked fashion.

### DNA fragmentation ELISA

A cellular DNA fragmentation ELISA was used to determine cell death quantitatively (Roche Diagnostics, Mannheim, Germany). This ELISA detects apoptotic fragmentation in BrdU-labeled DNA. Within 24 h after the last injection of either etanercept or the solvent, the assay was performed as described [17]. Briefly, the animals received a single i.p. injection of 20 mg/kg BrdU. Retinae were excised 24 h after injection of the BrdU, lysed in buffer (4 M urea,100 mM Tris, 20 mM NaCl, 200 mM EDTA, pH 7.4), and incubated with 4 mg/ml proteinase K at 55 °C. Genomic DNA fragments were isolated using the Apoptotic DNA ladder kit (Roche Diagnostics, Mannheim, Germany) according to the manufacturer’s instructions and finally eluted in 100 µl of elution buffer per retina. The amount of fragmented DNA that corresponded to each eluate, and therefore to each retina, was subsequently quantified using the DNA fragmentation ELISA (Roche Diagnostics) according to the manufacturer’s instructions. Photometric readings were obtained at 450 nm.

### Terminal transferase dUTP nick end labeling and M30 staining

Whole eyes from normal and diabetic rats treated with either etanercept or vehicle were fixed in 4% paraformaldehyde overnight at 4 °C and embedded in paraffin using standard histological procedures. Terminal Transferase dUTP nick end labeling (TUNEL) staining was performed in tissue sections as previously described [17,23,24] using the TUNEL kit (Roche Diagnostics). Briefly, DNA fragments in the tissue sections were labeled with digoxigenin-nucleotide and then allowed to bind an anti-digoxygenin antibody conjugated to a rhodamine reporter molecule. Apoptotic cells were detected using a CD-330 charge-coupled device (CCD) camera (Dage-MIT, Michigan, IN) attached to a Leica MZ FLIII microscope (Leica Microsystems, Deerfield, IL). The images were captured on an Apple (Cupertino, CA) G4 Computer and analyzed using Openlab software (Improvision, Lexington, MA). The number of positive endothelial cells was quantified by calculating the ratio of positive endothelial cells to negatively stained endothelial cells in retinal vessels. This was done in each of 12 sections from different eyes and expressed as percentage of positive cells.

Cytokeratin 18 is cleaved by caspases in early apoptotic events. The M30 CytoDeath antibody (Boehringer Mannheim) is a monoclonal mouse IgG2b antibody that binds to a caspase cleaved formalin resistant epitope of the cytokeratin 18 (CK18) cytoskeletal proteins. Immunoreactivity of the M30 antibodies is confined to the cytoplasm of apoptotic cells. It allows the determination of caspase-related enzymatic activity early in the course of apoptosis.

M30 Cytodeath immunoreactivity was evaluated as described [17,25]. In brief, 5 μm paraffin sections were deparaffinised, rehydrated, micro waved for 5 min in Dako antigen retrieval solution (Carpinteria, CA), treated for 30 min in methanol containing 0.5% H_2_O_2_, and then incubated for 1 h in incubation buffer (PBS containing 1% BSA and 0.1% Tween-20). The M30 CytoDeath antibody was applied for 1 h at room temperature in a 1:50 dilution in incubation buffer. The sections were subsequently washed in PBS containing 0.1% Tween-20 and incubated with an anti-fluorescein-POD (horse-radishperoxidase) antibody (Roche Diagnostics) according to the manufacturer’s instructions. The peroxidase reaction was developed with DAB (3,3-diaminobenzidine tetrahydrochloride), and the slides were counterstained with hematoxylin.

### Western blot analysis for caspase 3 and caspase 8

The levels of protein expression of caspase 3 and caspase 8 were evaluated by western blotting, performed as described previously [25,26]. Briefly, whole retinas were lysed for 30 min on ice in lysis buffer (50 mM Tris-HCl, pH 8, with 120 mM NaCl and 1% NP-40), supplemented with a mixture of proteinase inhibitors (Complete Mini; Roche Diagnostics). The samples were cleared by microcentrifugation (13,800x g, 30 min, 4 °C) and assessed for protein concentration. Thirty micrograms of protein per sample was electrophoresed in a 12% sodium dodecyl sulfate (SDS)-polyacrylamide gel (SDS–PAGE), and electroblotted onto nitrocellulose membranes. After a 1 h incubation in blocking solution (20% IgG-free normal horse serum, in PBS), the membranes were exposed overnight at 4 °C to an anti-caspase-3 antibody (Santa Cruz Biotechnology, Heidelberg, Germany) or an anti-caspase-8 antibody (Upstate Biotechnology, Lake Placid, NY). After washing in PBS, the respective secondary peroxidise-labeled antibody was applied at 1:10,000 dilution for 1 h at room temperature. The proteins were visualized with the enhanced chemiluminescence technique (ECL kit, Amersham Pharmacia Biotech, Piscataway, NJ). Protein loading was assessed by β-actin immunostaining and Coomassie staining of the membrane.

### Enzymatic activity of caspase 3 and caspase 8

The activity of caspase 3 and caspase 8 was measured by a colorimetric analysis of the enzymatic conversion of a specific substrate as described [17]. The activity of caspases 3 and 8 was measured via the enzymatic conversion of a specific substrate. In brief, retinas were lysed in a buffer containing 20 mM PIPES, 100 mM NaCl, 10 mM DTT, 1 mM EDTA, 0.1% CHAPS, and 10% sucrose, pH 7.2. After centrifugation of the homogenates at 13,800x g rpm for 10 min, 40 µl aliquots of the supernatants were pipetted into 96 well plates. Excess amounts (80 µl) of caspase 8 substrate (Bachem Biochemica GmbH, Heidelberg, Germany) or caspase 3 substrate (Alexis Biochemicals, Grünberg, Germany) were added to the supernatants. The reaction product was quantified by fluorophotometry on a plate reader (Beckmann, Hamburg, Germany) at 480 nm after 1, 2, 5, and 15 min (caspase 3) or 1, 60, 120, and 180 min (caspase 8). The results were normalized to total protein per sample as determined by the Bradford assay (Bio-Rad, Munich, Germany).

### Trypsin digests of the retinal tissue

Trypsin digestion of the retina was performed according to the method of Cogan et al. [27] with some modification [19,28]. Formaldehyd-fixed retinas were divided at the optic disk into four quadrants. The retinal tissue was incubated in 3% Trypsin, 0.1 M Tris (pH 7.8) for 1–3 h at 37 °C until the medium became cloudy and the tissues showed signs of disintegration. The retinas were than transferred to water and shaken gently to free the vessel network from adherent retinal tissue, washed in fresh water, mounted on glass slides and allowed to dry. They were stained with hematoxylin. Endothelial cells have large, ellipsoid nuclei lying within the vessel wall along the axis of the capillaries whereas pericytes nuclei are smaller, darker and usually round nuclei situated on the outer portion of the vessel wall. Mice that were diabetic for 11, 16, or 20 months were used. A digital imaging system was used to analyze four quadrants per retina for histopathological changes, including acellular capillary microaneurysm formation, and these were then grouped together. Four retinas per group were analyzed. All measurements were performed in a masked fashion.

### Statistical analysis

All results are expressed as the mean**±**standard deviation (SD). The data were compared by ANOVA with post hoc comparisons tested by using Fisher’s protected least significant difference procedure. The data of the quantitative PCR were analyzed by Whitney-Mann-U-test. Differences were considered statistically significant when p values were less than 0.05.

## Results

### Etanercept reduces diabetes-induced retinal endothelial cell injury

**Figure 1:**
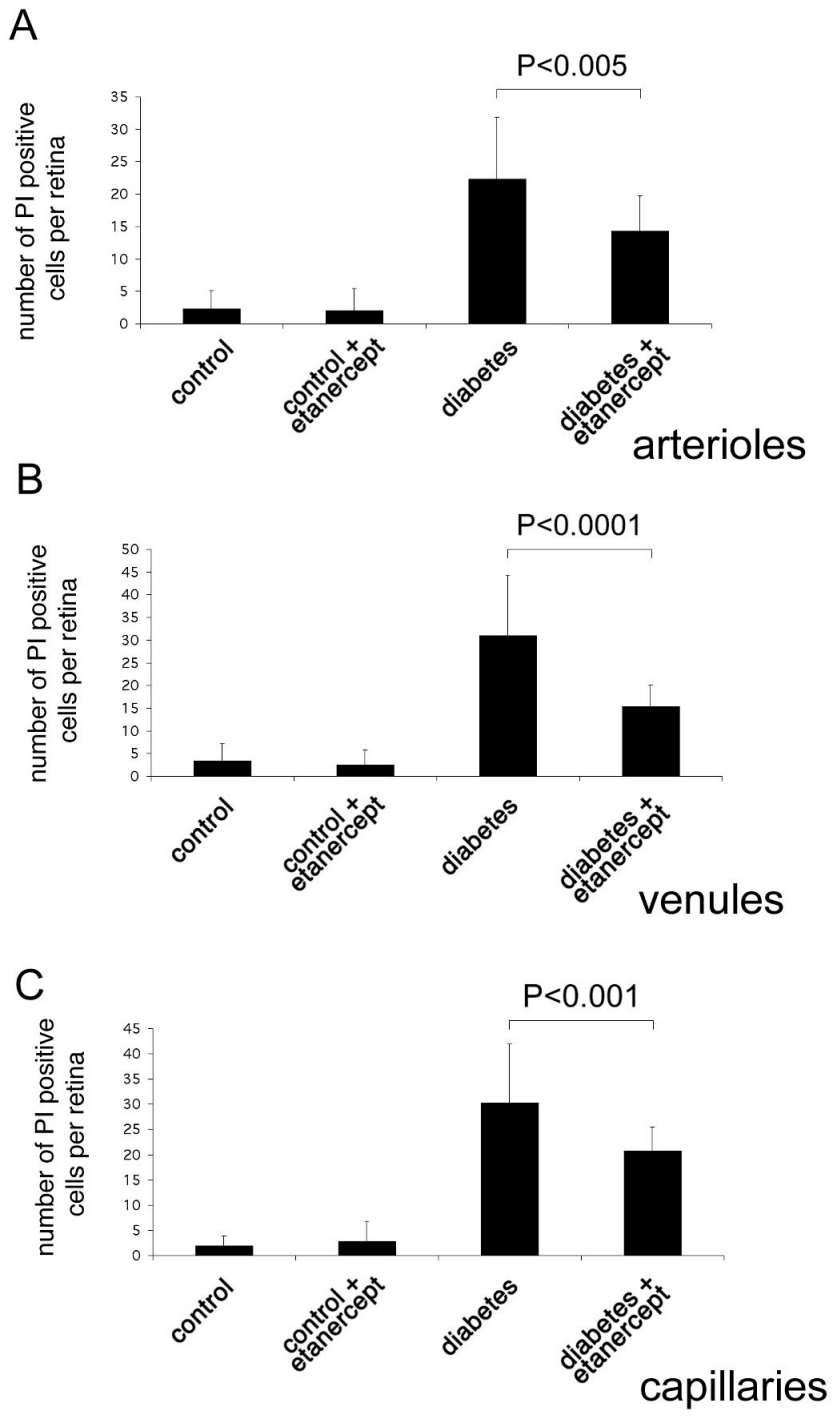
Quantification of endothelial cell injury and death by propidium iodide staining. Dead and injured retinal endothelial cells were labeled in vivo using propidium iodide. Endothelial cell damage was increased in diabetic animals in capillaries (**A**), venules (**B**), and arteries (**C**). Administration of the TNF-α inhibitor etanercept reduced diabetes-induced endothelial cell injury in all vessel types.

To investigate the role of TNF-α in mediating retinal endothelial cell death in early diabetic retinopathy, we assessed the effect of etanercept on retinal endothelial injury using a PI labeling assay as previously described [2]. According to our previously published data, two weeks after induction of diabetes the amount of PI-labeled cells increased significantly compared to nondiabetic controls. PI-positive cells were mainly located in clusters, which were found in the capillaries, venules, and arterioles (Figure 1). The total number of PI-positive cells per retina was 7.46±7.14 (n=13) in nondiabetic control animals compared to 83.54±16.02 (n=13; p<0.0001) in diabetic animals. Treatment of diabetic animals with etanercept resulted in a 39.5% reduction of PI-positive cells (50.46±9.48 PI positive cells per retina; n=13; p<0.0001) compared to diabetic animals without treatment. In nondiabetic animals, there was no significant change in the number of PI-positive cells after treatment with etanercept (7.23±7.00 PI positive cells per retina; n=13, p>0.05).

### Etanercept reduces diabetes-induced apoptosis in the retina

**Figure 2:**
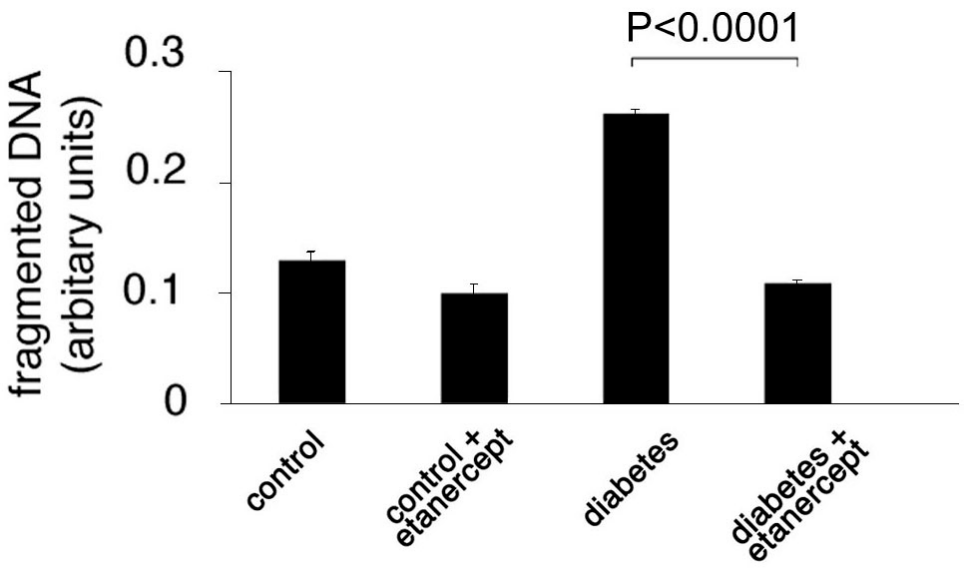
Cellular DNA fragmentation ELISA in the retina. DNA fragmentation and therefore apoptosis was significantly increased in the retinal tissue of diabetic compared to nondiabetic animals. Treatment with etanercept reduced the diabetes-induced increase in DNA fragmentation.

To quantify the apoptotic cell death in the retina, we used a modified ELISA for fragmented DNA (Figure 2). Two weeks after induction of diabetes with STZ, fragmented DNA had increased twofold (0.13 ± 0.01 versus 0.26±0.07 arbitrary units; n=6; p<0.0001) when compared to the controls. Systemic treatment with the TNF-α inhibitor etanercept reduced fragmented retinal DNA by half (0.11±0.01; n=6; p<0.0001), resulting in levels comparable to the nondiabetic controls.

### Etanercept reduces diabetes-induced retinal endothelial cell apoptosis

**Figure 3:**
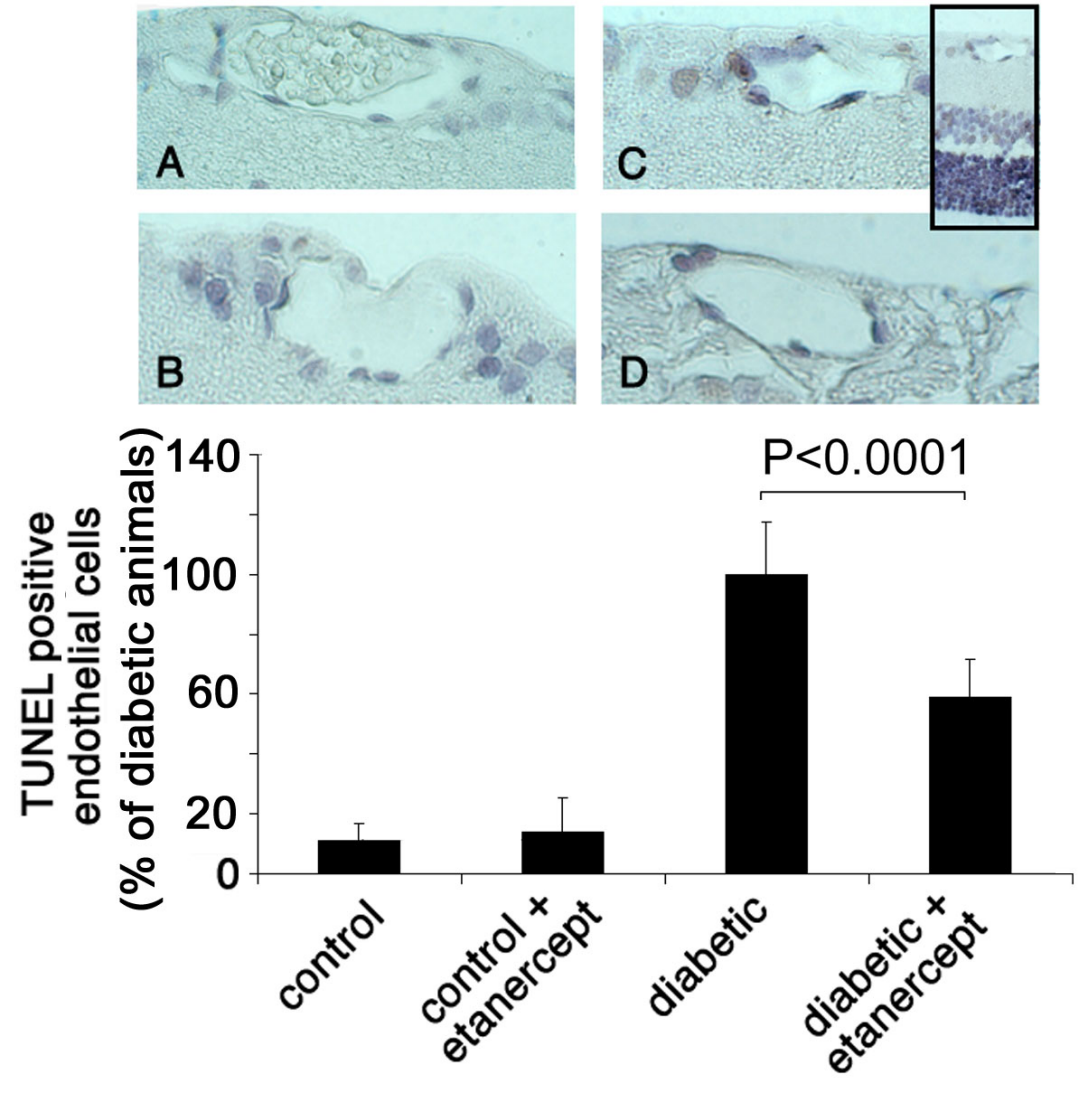
TUNEL staining of retinal sections. Paraffin sections of eyes from nondiabetic rats (**A**), nondiabetic rats treated with etanercept (**B**), diabetic rats (**C**), and diabetic rats treated with etanercept (**D**) were assessed by TUNEL. Sections were subsequently counterstained with hematoxylin. Insert shows an overview of a diabetic retina with the ganglion cell layer (above), the inner nuclear layer (middle, light blue) and the outer nuclear layer (below, dark blue). Numbers of positive endothelial cells were counted in each of 12 sections and expressed as percent of total endothelial cells. Treatment with etanercept reduced diabetes-induced endothelial cell apoptosis, as detected by TUNEL staining.

**Figure 4:**
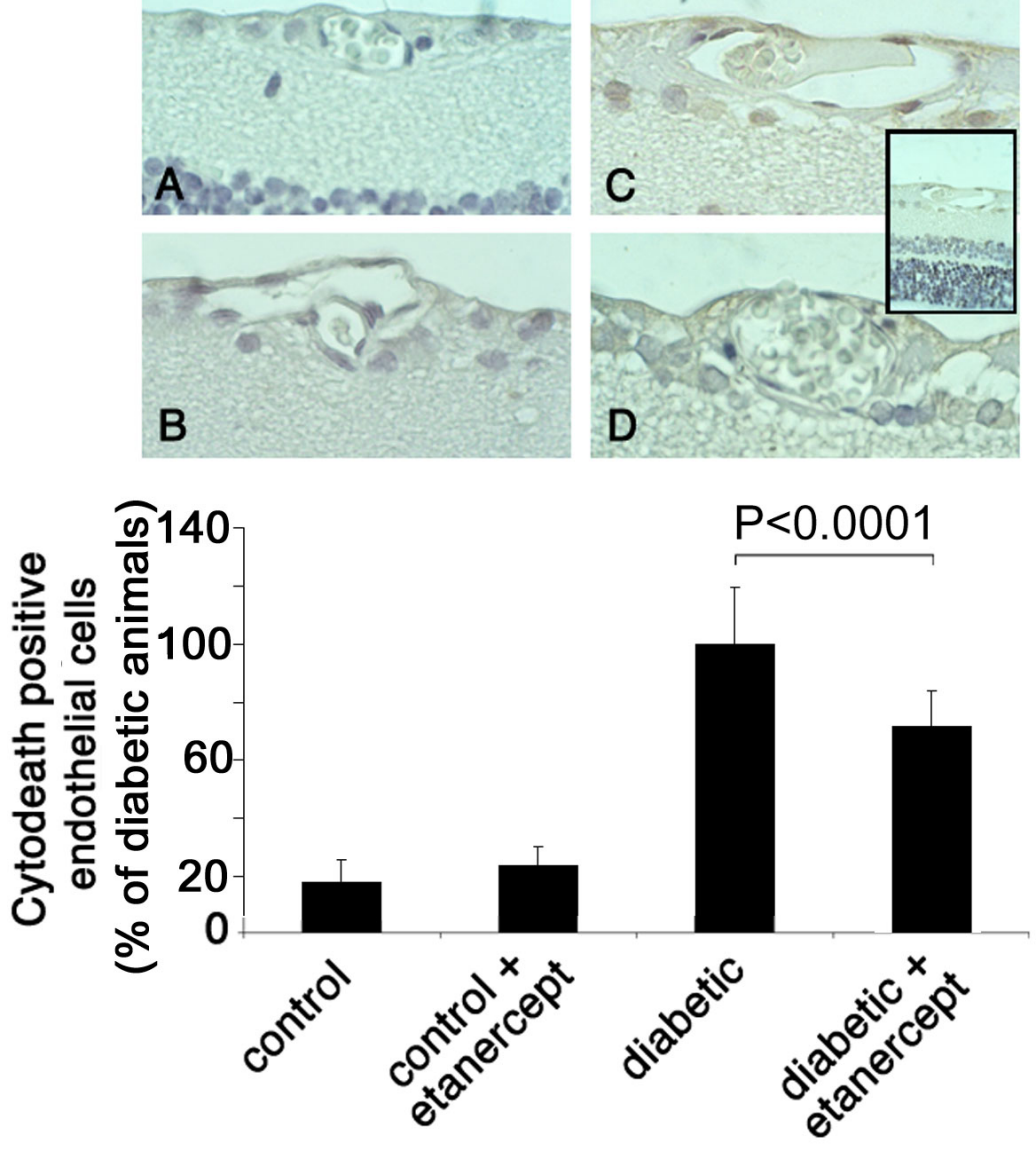
M30 CytoDeath staining of retinal sections. Paraffin sections of eyes from non-diabetic rats (**A**), non-diabetic rats treated with etanercept (**B**), diabetic rats (**C**), and diabetic rats treated with etanercept (**D**) were assessed with M30 staining. Sections were subsequently counterstained with hematoxylin. Insert shows an overview of a diabetic retina with the ganglion cell layer (above), the inner nuclear layer (middle, light blue) and the outer nuclear layer (below, dark blue). Numbers of positive endothelial cells were counted in each of 12 sections and expressed as percent of total endothelial cells. Treatment with etanercept reduced endothelial cell apoptosis and caspase activity as determined by M30 staining.

To localize the apoptotic cell death within the retina, we stained formalin-fixed, paraffin-embedded retinal sections by using TUNEL assay (Figure 3) and M30 CytoDeath antibody (Figure 4). TUNEL staining labels fragmented DNA that is localized in the nucleus, whereas M30 stains caspase-cleaved cytokeratins that are located in the cytoplasm. Almost no TUNEL positivity or M30 staining was detected anywhere in the retina in nondiabetic animals with or without etanercept treatment (Figure 3 and Figure 4). In two-week diabetic animals, TUNEL-positive and M30-positive cells were located predominantly in the vascular endothelium of the retinal vessels as well as in the inner nuclear layer. Few positive cells were found in the ganglion layer. Treatment with etanercept was able to reduce TUNEL and M30 staining throughout the retina in diabetic animals (Figure 4).

To quantify the amount of apoptotic cell death in the retinal vessels, we determined the ratio of TUNEL-positive endothelial cells or M30-positve cells to the total number of endothelial cells in cross-sections of 12 distinct eyes from each of six animals per group. After two weeks of diabetes, the ratio of TUNEL-positive cells increased 6.2 fold when compared to nondiabetic control animals (n=12; p<0.0001). Treating diabetic animals with etanercept reduced the TUNEL-positive staining by one-third (n=12; p<0.0001; Figure 3). Similarly, M30 staining was rare in the endothelial cells of nondiabetic control animals, however, the ratio of M30-positve cells increased 8.9 fold after a two-week duration of diabetes (n=12; p<0.0001). The percentage of M30-positive endothelial cells was reduced by 41% (n=12; p<0.0001) after inhibition of TNF-α activity with etanercept (Figure 4). These results correlate with the data analyzing PI positivity as demonstrated in the previous section.

### Inhibited caspase activation by Etanercept in diabetic retinopathy

**Figure 5:**
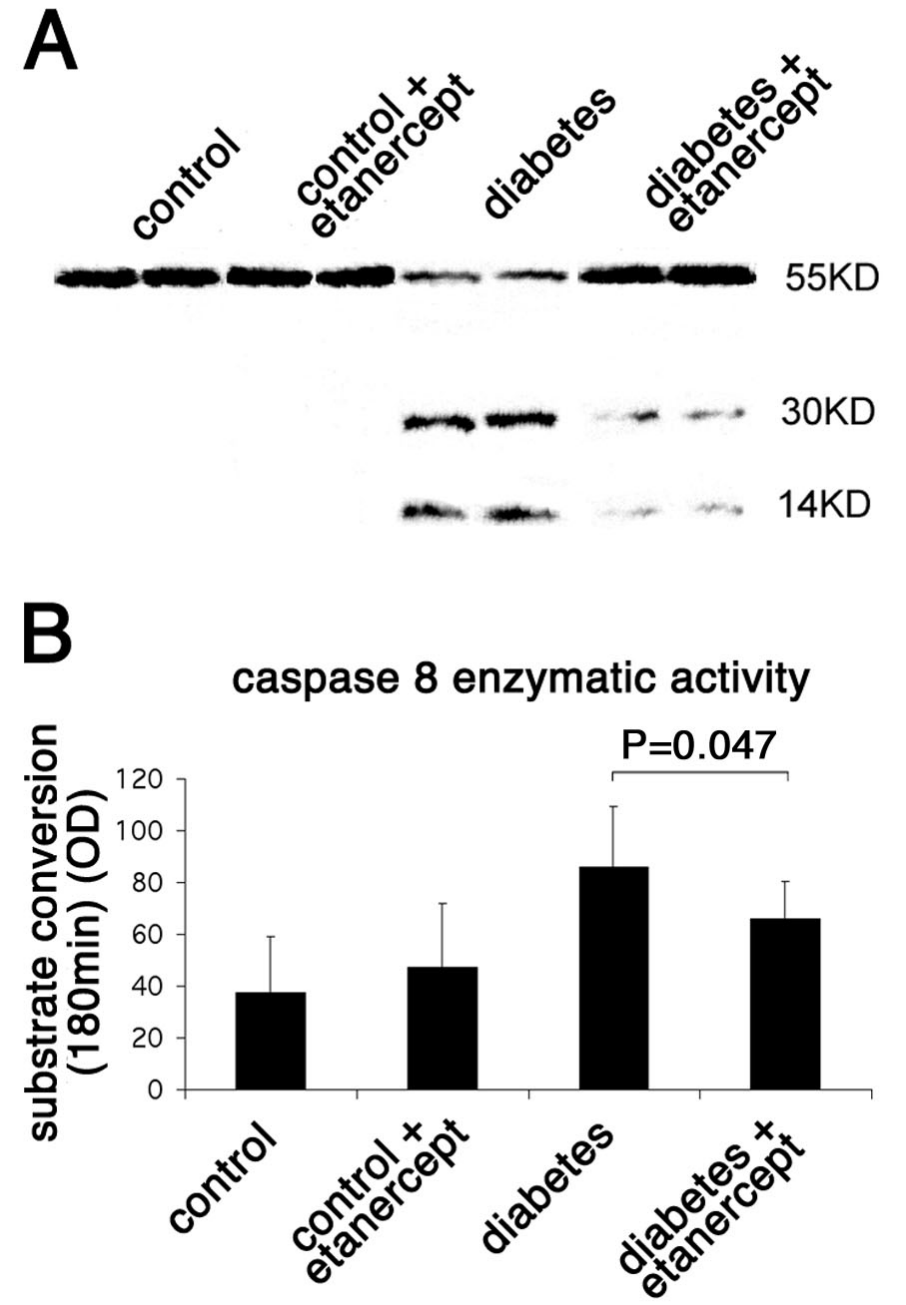
Caspase 8 enzymatic activity in rat retinas. **A:** The level of protein expression of caspase 8 in the rat retina was evaluated by western blotting. Diabetes increased the cleaved form of caspase 8 (activated form), whereas treatment with etanercept reduced the diabetes-induced cleavage of caspase 8. **B:** The enzymatic activity of caspase 8 in the rat retina was evaluated by a colorimetric analysis. Diabetes increased the activity of caspase 8 as measured by the conversion of its specific substrate. Treatment with etanercept reduced the diabetes-induced caspase 8 activation and therefore the conversion of its substrate and correlates with the reduction in the cleaved form of the caspase as measured by the western blot shown.

**Figure 6:**
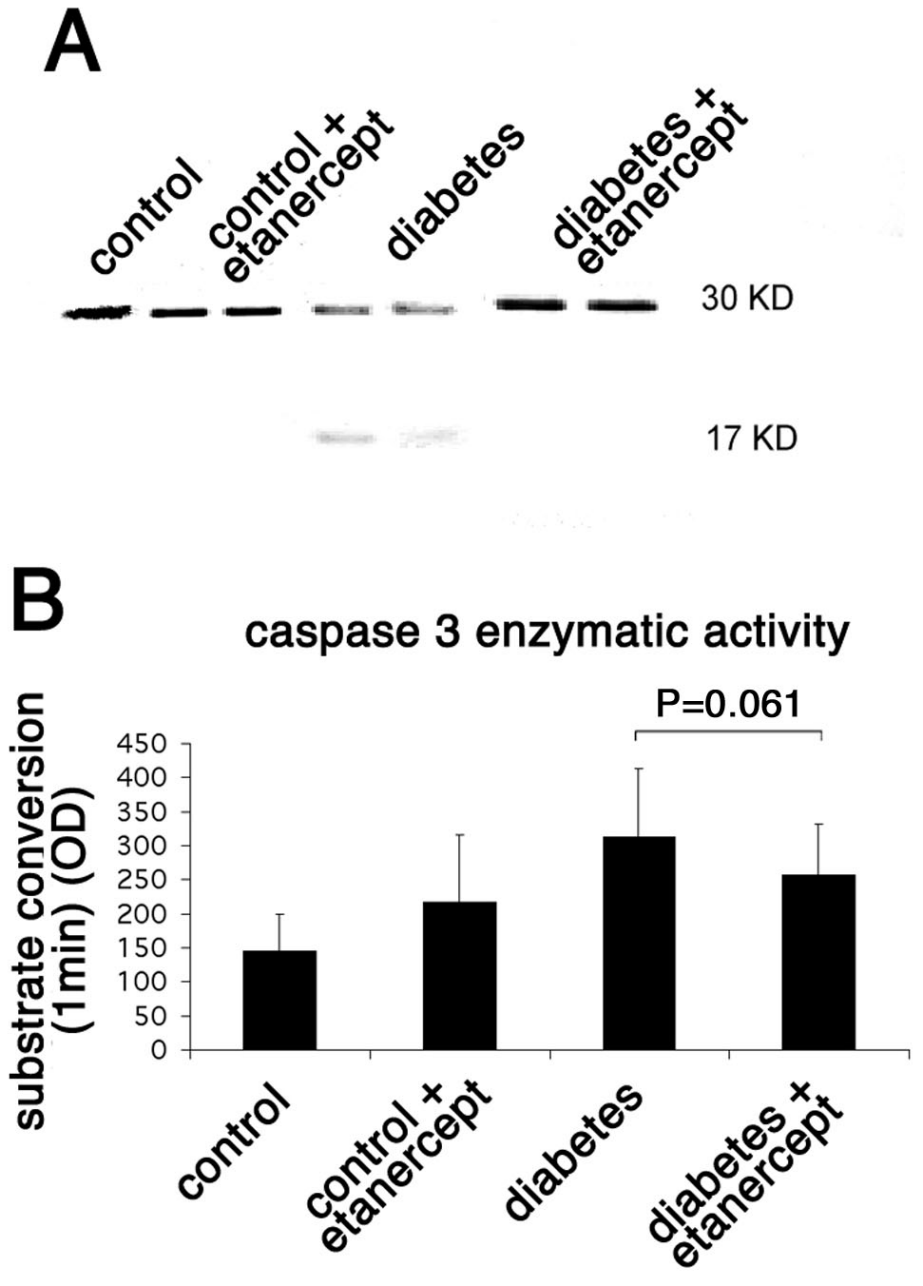
Caspase 3 enzymatic activity in rat retinas. **A:** The level of protein expression of caspase 3 in the rat retina was evaluated by western blotting. Diabetes increased the cleaved form of caspase 3 (activated form), whereas treatment with etanercept reduced the diabetes-induced cleavage of caspase 3. **B:** The enzymatic activity of caspase 3 in the rat retina was evaluated by a colorimetric analysis. Diabetes increased the activity of caspase 3 as measured by the conversion of its specific substrate. Treatment with etanercept reduced the diabetes-induced caspase 3 activation and therefore the conversion of its substrate and correlates with the reduction in the cleaved form of the caspase as measured by the western blot shown.

The increase in M30 staining observed in diabetic retinas suggested the involvement of caspases in mediating apoptotic cell death. During apoptosis, caspases are activated in a hierarchical manner to cleave structural and functional proteins needed for cell survival. Cleaved products for both caspase 8 and caspase 3 were found by western blot analysis in the retinal lysates of rats after two weeks of diabetes, proving the activation of these caspases in the diabetic retina in the rat (Figure 5 and Figure 6). Administration of the soluble TNF-α inhibitor in the diabetic rats significantly reduced the cleaved forms for both caspase 8 (14 kDa band; Figure 5) and caspase 3 (17 kDa band; Figure 6), an observation that correlates with the reduced levels of apoptotic cells in these rats as described above. Furthermore, the ability of these caspases to cleave their specific substrates was tested directly in the retinal lysates. Caspase 3 was found to cleave its substrate efficiently as early as 1 min after incubation. In comparison to nondiabetic animals, the substrate conversion rate was 2.16-fold higher in the diabetic retinas (322.69±99.71 versus 149.73±54.51 arbitrary units; n=3; p<0.001). Treatment of diabetic animals with etanercept reduced the conversion rate to 262.01±74.9 (n=3; p=0.061).

Caspase 8 activity was measured 180 min after incubation with the substrate. In comparison to nondiabetic animals, the substrate conversion rate was 2.28 fold higher in the diabetic retinas (37.63 ± 21.6 versus 85.80 ± 23.7 arbitrary units; n=3; p<0.0005). Treatment of diabetic animals with etanercept reduced the conversion rate to 65.94 ± 14.35 (n=3; p=0.047).

### Histopathological capillary changes in long-term galactose-fed mice

To achieve an extended observation period, we investigated galactose-fed mice, which demonstrate similar histopathological changes as diabetic animals [19]. With galactose feeding we were able to analyze trypsin digests of the retinas in 11-month-, 16-month-, and 20-month-old galactosemic mice and age-matched controls fed with regular laboratory chow, respectively.

**Figure 7:**
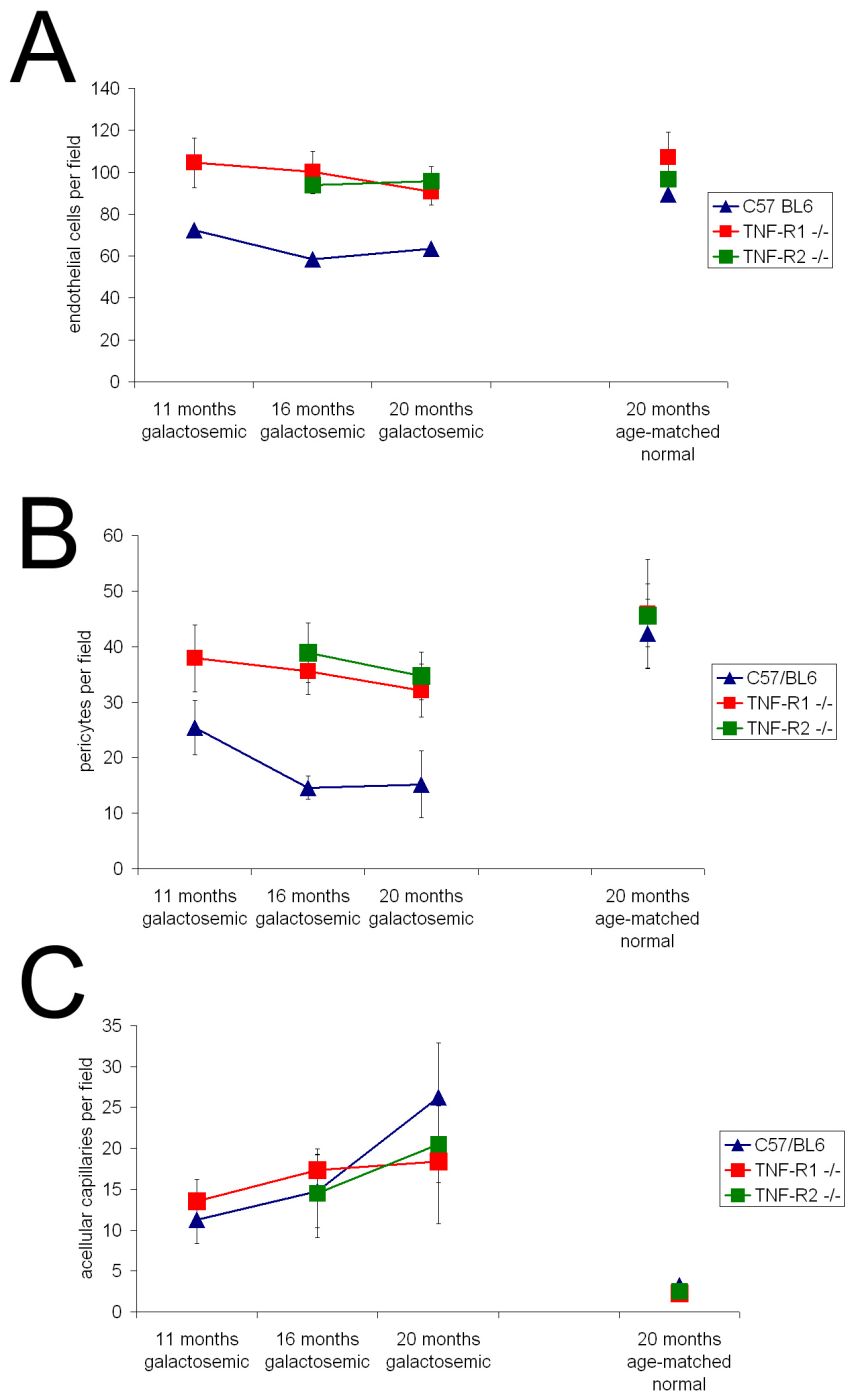
Retinal histopathological changes were investigated in galactosemic TNF-RI−/− and TNF-RII−/− mice and their respective wild-type controls. Both the number of endothelial cells per field as well as the number of pericytes per field significantly decreased throughout the course of the disease in the wild-type galactosemic animals (**A, B**). The number of acellular capillaries was significantly increased at 20 months of galactosemia (**C**).

Hypergalactosemia reduced the number of endothelial cells in all of the three mouse strains (Figure 7 A). Endothelial cells decreased by 15% after 11 months, by 7% after 16 months and by 38% after 20 months in hypergalactosemic wild-type mice as compared to non-galactosemic controls.

However, TNF-α receptor deficiency protected against endothelial cell loss. In both galactosemic Rp55^−/−^ and Rp75^−/−^ mice the number of endothelial cells was significantly higher than in galactosemic wild-type mice as counted after 15–16 months (100.2±9.8 and 93.8±4.0 versus 58.6±18.82; p<0.001) and 18–20 months (90.6±6.3 and 95.8±6.8 versus 64,4±26.1; p<0.05) of hypergalactosemia. After 20 months of galactosemia, endothelial cell counts in Rp55^−/−^ mice and Rp75^−/−^ mice did not differ significantly from age-matched normal litter mates (90.6±6.3 versus 107±12.3 and 95.8+6.8 versus 96.6±7.2 (p>0.05).

The number of pericytes decreased in all Rp55^−/−^, Rp75^−/−^, and wild-type mice during the time course of hypergalactosemia (Figure 7B). Maximal pericyte reduction reached 30%, 23.9%, and 66.4% after 20 months. Again, cell loss was smaller in TNFR-deficient mice when compared to the wild-type mice. While no significant difference could be detected between Rp55^−/−^ and Rp75^−/−^ mice, both TNF-deficient mouse strains showed less pericyte loss than wild-type mice. The differences in number of pericytes between the three groups were significant after both 16 months (35.6±4.2 and 38.9±5.4 versus 14.6±2.1; p<0.001) and 20 months (32.1±4.8 and 34.7±4.3 versus 15.2±6.0; p<0.01).

**Figure 8:**
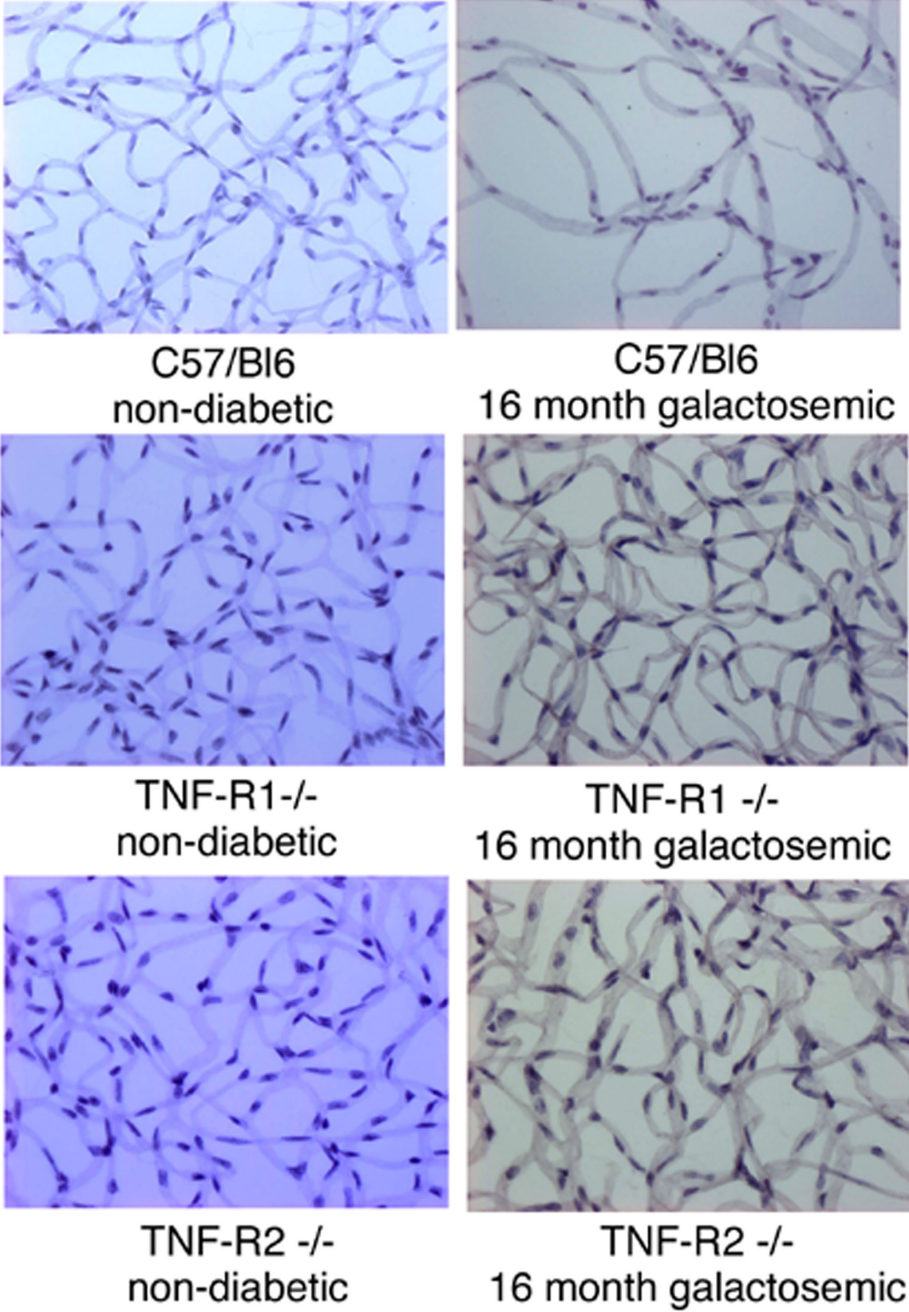
Representative images of the capillary bed in galacosemic animals and age-matched controls. Histopathological changes were investigated in galactosemic TNF-RI^−/−^ and TNF-RII^−/−^ mice and their respective wild-type controls by trypsin digestion of the retinas. Representative images of the capillary bed of trypsin digested retinas in age-matched controls (left column) and galactosemic animals (right column) are shown.

Furthermore, the formation of acellular capillaries per retina was quantified as described previously [4,6,19,28]. In all groups of animals fed with normal chow, five or fewer acellular capillaries per retina were found: C57/BL6 3.25±0.5, Rp55^−/−^ 2.25±0.5, and Rp75^−/−^ 2.5±0.57. In all strains, the number of acellular capillaries increased with the duration of galactose feeding. The number of acellular capillaries showed an approximately ninefold increase after 20 months of hypergalactosemia (Figure 7C) and a decrease in TNFR-deficient mice. Representative images of the capillary bed of trypsin digested retinas in galactosemic animals and age-matched controls are presented in Figure 8. The loss of endothelial cells and pericytes as well as the acellular capillaries in the retinas of 16 months galactosemic animals can be seen clearly.

## Discussion

In the current study, we investigated the role of the inflammatory cytokine TNF-α in the apoptotic cell death of retinal endothelial cells during early and late stages of diabetic retinopathy using a rat model of streptozotocin-induced diabetes and a mouse model of long-term galactosemia.

Our data emphasize the role of TNF-α in signature pathologies in both early and late diabetic retinopathy. We have previously demonstrated that TNF-α increases early during the course of diabetic retinopathy [14]. TNF-α is a pro-inflammatory cytokine produced mainly by monocytes, macrophages, activated endothelial cells, fibroblasts, and joint cartilage chondrocytes [11]. The induction of TNF-α could be attributed to various conditions that operate in the course of diabetes, such as hyperglycemia and oxidative stress, and inflammatory cytokines such as VEGF or interleukins, which activate the transcription factor NF-κB [29,30]. We have previously demonstrated that treatment with high doses of antiinflammatory agents, such as aspirin and the selective cyclooxygenase-2 (COX-2) inhibitor meloxicam, reduces the levels of TNF-α in the diabetic retina, proving that inflammatory mediators and COX-2 are central in the upregulation of this cytokine [14].

Endothelial and pericyte cell loss is one of the earliest and key manifestations of diabetic retinopathy. It leads to blood-retinal barrier breakdown, formation of acellular capillaries, microaneurysms, and retinal ischemia that hallmark the transition to proliferative diabetic retinopathy. As we [17] and others [31-34] have previously described, during the course of diabetes, injury and apoptosis occur early in endothelial cells, retinal ganglion cells, and astrocytes. Singh et al. detected apoptosis in endothelial cells in microvessels three days after the induction of diabetes [31], and Emanueli et al. have shown that in the STZ model, endothelial cells die via apoptosis as early as two weeks after the induction of diabetes [32]. Mizutani et al. noted accelerated death of endothelial cells in the retinas from diabetic patients [33], and Barber et al. demonstrated increased neural cell death as early as one month after the induction of diabetes in male Sprague-Dawley rats [34]. In the current study, we found increased amounts of DNA fragmentation in diabetic versus non-diabetic retinas, confirming the increased incidence of apoptosis, and we localized this apoptotic activity in the endothelial, pericyte, and ganglion cell layer. The same areas demonstrated staining with the M30 antibody, indicating caspase activation, and resulting in apoptotic death.

Caspases, a family of cysteine proteases, are both involved in activation of cytokines as well as in the initiation and execution of apoptosis [35]. Initiator caspases such as caspase 8, are known to activate proteolytically executioner caspases, such as caspase 3 [36,37]. The activation of both the initiator as well as the effector caspases in complete retinal extracts was also shown. Our results correlate with Martin and coworkers, who found activation of caspase 3 in cells of the ganglion cells layer in diabetic retinas in a statistically higher degree than in retinas from nondiabetic controls in the STZ mouse model [38].

According to our data, all the aforedescribed pathologies, including cellular loss, TUNEL reactivity, and caspase activation are significantly reduced in the anti-TNF-α-treated retinas. This leads us to the conclusion that TNF-α is a major player in retinal cell death in diabetic retinopathy. TNF-α is known to be a potent inducer of apoptosis in endothelial cells [15,16]. TNF signal transduction is mediated through two cell surface receptors, TNF-RI and TNF-RII. Until recently, it was believed that TNF-induced apoptosis was mediated exclusively by TNF-RI because TNF-RII lacks the death domain. However, it has been demonstrated that TNF-RII enhances TNF-RI-mediated apoptosis. The significant inhibition of the diabetes-induced activation of caspase 8 and 3 in our model leads to the conclusion that TNF-α induces retinal cell death in diabetes through these two caspases.

However, there might be also an indirect involvement for TNF-α in diabetic apoptotic cell death. In experimental diabetes, adhesion of leukocytes to the retinal vasculature is one of the earliest events and results in enhanced blood-retinal barrier breakdown, endothelial injury, and cell death as well as capillary nonperfusion [1,2]. As we have previously shown, leukocyte-induced apoptosis is mediated by the Fas-FasL pathway [17,23], and administration of soluble etanercept reduced leukocyte adhesion [14]. Since sequestration of TNF-α reduces retinal apoptosis, it is justified to hypothesize that TNF-α could induce apoptosis indirectly by increasing leukocyte adhesion. TNF-α has been shown to increase NF-κB in many models, whereas NF-κB is a known regulator of FasL expression. It is interesting to speculate that the early rise in TNF-α expression in diabetes results in activation of NF-κB that in turn upregulates FasL expression in leukocytes [39]. Subsequently, these leukocytes are better equipped to induce Fas-mediated apoptosis in their targets as demonstrated previously [17]. During inflammation, TNF-α and VEGF induce endothelial cell downregulation of FasL and upregulate Fas and adhesion molecules such as ICAM-1 [40]. This downregulation of FasL in the endothelium, combined with the increased apoptotic threshold of leukocytes in diabetes [41], could result in a relative increase of surviving leukocytes in immunoprivileged areas such as the retina. In addition, the converse upregulation of adhesion molecules and Fas on the endothelium, and increases in FasL on the leukocytes, could function together to drive the balance toward a significantly increased leukocyte-induced endothelial death in diabetes. This effect might be mediated by circulating factors such as VEGF, TNF-α, or oxidative stress. The activation of caspase 8 and caspase 3 in retinas of diabetic rats according to our results and the reduction of this activation by inhibition of TNF-α activity fit in the model of a TNF-α initiated receptor-mediated apoptosis.

In this study, apoptosis was significantly reduced in endothelial cells as well as in the inner nuclear layer in the retinas of diabetic rats upon the administration of the TNF-α inhibitor and the concomitant reduction of leukocyte adhesion. This leads to the conclusion that leukocytes that adhere to the vasculature might be responsible for the observed endothelial apoptosis, whereas leukocytes extravasating to the retinal tissue are responsible for the apoptotic death observed in other cell layers. This is in accordance with our previous data in diabetes, where inhibition of leukocyte-mediated death by neutralizing FasL reduced apoptotic death not only in the endothelial cell layer but also in the ganglion and inner cell layer [17].

To establish a role for TNF-α in the pathogenesis of signature lesions in diabetic retinopathy and to address the question whether the demonstrated effects are only due to its transient overexpression in early diabetes, we used a long-term mouse model of diabetic retinopathy. The course of diabetes in rodents includes the formation of signature lesions similar to those in humans, such as acellular capillary formation, microaneurysms breakdown of the blood-retinal barrier, and macular edema. These similarities allow extrapolating valuable conclusions from the rodent models of long-term diabetes relevant to human diabetes. We have examined the vascular pathology in a mouse model of galactose-induced diabetes that allows for the analysis of the histopathological alterations up to two years after the induction of diabetes. We [19] and others [4] have found that throughout the course of experimental galactosemia the number of endothelial cells and pericytes decreased significantly, whereas the formation of acellular capillaries and microaneurysms increased [33]. Pericyte loss is in the center of the pathogenesis of diabetes, allowing for microaneurysm formation possibly by local weakening and out-pouching of the capillary wall, and contributing to the endothelial dysfunction and damage that leads to cellular death and formation of acellular capillaries. Surprisingly enough, the formation of acellular capillaries was not reduced in the TNF-RI- and TNF-RII-deficient mice although the pericyte and endothelial cell loss was significantly inhibited. This observation could lead to the conclusion that other factors apart from the net cellular loss contribute to the formation of acellular capillaries.

In conclusion, our data indicate that the proinflammatory cytokine TNF-α plays a major role in endothelial and retinal cell injury and apoptosis during diabetes. TNF-α is involved in endothelial apoptosis directly via death receptor-mediated apoptosis and indirectly via an increased leukostasis. Together with our previous data demonstrating the role of TNF-α on diabetic vascular leakage [14], we can conclude from this study that TNF-α is one of the major cytokines in diabetic retinopathy both involved in leukocyte activation as well as in endothelial cell apoptosis. The inhibition of both initiation- and execution-caspase activation in the diabetic model by etanercept demonstrates the potential of the TNF-α antagonist to inhibit diabetic apoptosis, including the death-inducing signaling complex. However, the exact death receptor pathways either triggered by FAS (CD95) or via TNF-RI remain to be elucidated. Given their established efficacy and safety profile, biologic agents that neutralize TNF-α have received approval from the USA Food and Drug Administration for the treatment of rheumatoid arthritis [42]. These drugs could be of potential benefit in preventing early diabetic vascular damage.

## References

[1] Miyamoto K Khosrof S Bursell SE Rohan R Murata T Clermont AC Aiello LP Ogura Y Adamis AP Prevention of leukostasis and vascular leakage in streptozotocin-induced diabetic retinopathy via intercellular adhesion molecule-1 inhibition. Proc Natl Acad Sci USA 1999 96 10836 41 10485912 10.1073/pnas.96.19.10836 PMC17969

[2] Joussen AM Murata T Tsujikawa A Kirchhof B Bursell SE Adamis AP Leukocyte-mediated endothelial cell injury and death in the diabetic retina. Am J Pathol 2001 158 147 52 11141487 10.1016/S0002-9440(10)63952-1 PMC1850259

[3] Engerman RL Kern TS Retinopathy in animal models of diabetes. Diabetes Metab Rev 1995 11 109 20 7555563 10.1002/dmr.5610110203

[4] Kern TS Engerman RL Galactose-induced retinal microangiopathy in rats. Invest Ophthalmol Vis Sci 1995 36 490 6 7843917

[5] Kern TS Engerman RL A mouse model of diabetic retinopathy. Arch Ophthalmol 1996 114 986 90 8694735 10.1001/archopht.1996.01100140194013

[6] Kern TS Engerman RL Capillary lesions develop in retina rather than cerebral cortex in diabetes and experimental galactosemia. Arch Ophthalmol 1996 114 306 10 8600891 10.1001/archopht.1996.01100130302013

[7] Bazzoni F Beutler B The tumor necrosis factor ligand and receptor families. N Engl J Med 1996 334 1717 25 8637518 10.1056/NEJM199606273342607

[8] Kacimi R Karliner JS Koudssi F Long CS Expression and regulation of adhesion molecules in cardiac cells by cytokines: response to acute hypoxia. Circ Res 1998 82 576 86 9529162 10.1161/01.res.82.5.576

[9] Lim JW Kim H Kim KH Nuclear factor-kappaB regulates cyclooxygenase-2 expression and cell proliferation in human gastric cancer cells. Lab Invest 2001 81 349 60 11310828 10.1038/labinvest.3780243

[10] Chen CC Manning AM Transcriptional regulation of endothelial cell adhesion molecules: a dominant role for NF-kappa B. Agents Actions Suppl 1995 47 135 41 7540353 10.1007/978-3-0348-7343-7_12

[11] Wallach D Cell death induction by TNF: a matter of self control. Trends Biochem Sci 1997 22 107 9 9149526 10.1016/s0968-0004(97)01015-3

[12] Spranger J Meyer-Schwickerath R Klein M Schatz H Pfeiffer A. TNF-alpha level in the vitreous body. Increase in neovascular eye diseases and proliferative diabetic retinopathy. Med Klin (Munich) 1995 90 134 7 7723714

[13] Wright PS Cooper JR Kropp KE Busch SJ Induction of vascular cell adhesion molecule-1 expression by IL-4 in human aortic smooth muscle cells is not associated with increased nuclear NF-kappaB levels. J Cell Physiol 1999 180 381 9 10430178 10.1002/(SICI)1097-4652(199909)180:3<381::AID-JCP9>3.0.CO;2-F

[14] Joussen AM Poulaki V Mitsiades N Kirchhof B Koizumi K Dohmen S Adamis AP Nonsteroidal anti-inflammatory drugs prevent early diabetic retinopathy via TNF-alpha suppression. FASEB J 2002 16 438 40 11821258 10.1096/fj.01-0707fje

[15] Polunovsky VA Wendt CH Ingbar DH Peterson MS Bitterman PB Induction of endothelial cell apoptosis by TNF alpha: modulation by inhibitors of protein synthesis. Exp Cell Res 1994 214 584 94 7925652 10.1006/excr.1994.1296

[16] Robaye B Mosselmans R Fiers W Dumont JE Galand P Tumor necrosis factor induces apoptosis (programmed cell death) in normal endothelial cells in vitro. Am J Pathol 1991 138 447 53 1992769 PMC1886201

[17] Joussen AM Poulaki V Mitsiades N Cai WY Suzuma I Pak J Ju ST Rook SL Esser P Mitsiades CS Kirchhof B Adamis AP Aiello LP Suppression of Fas-FasL-induced endothelial cell apoptosis prevents diabetic blood-retinal barrier breakdown in a model of streptozotocin-induced diabetes. FASEB J 2003 17 76 8 12475915 10.1096/fj.02-0157fje

[18] Joussen AM Huang S Poulaki V Camphausen K Beecken WD Kirchhof B Adamis AP In vivo retinal gene expression in early diabetes. Invest Ophthalmol Vis Sci 2001 42 3047 57 11687554

[19] Joussen AM Poulaki V Le ML Koizumi K Esser C Janicki H Schraermeyer U Kociok N Fauser S Kirchhof B Kern TS Adamis AP A central role for inflammation in the pathogenesis of diabetic retinopathy. FASEB J 2004 18 14502 15231732 10.1096/fj.03-1476fje

[20] Barner A Review of clinical trials and benefit/risk ratio of meloxicam. Scand J Rheumatol Suppl 1996 102 29 37 8628979 10.3109/03009749609097228

[21] Moreland LW McCabe DP Caldwell JR Sack M Weisman M Henry G Seely JE Martin SW Yee CL Bendele AM Frazier JL Kohno T Cosenza ME Lyons SA Dayer JM Cohen AM Edwards CK III Phase I/II trial of recombinant methionyl human tumor necrosis factor binding protein PEGylated dimer in patients with active refractory rheumatoid arthritis. J Rheumatol 2000 27 601 9 10743796

[22] Moreland LW Schiff MH Baumgartner SW Tindall EA Fleischmann RM Bulpitt KJ Weaver AL Keystone EC Furst DE Mease PJ Ruderman EM Horwitz DA Arkfeld DG Garrison L Burge DJ Blosch CM Lange ML McDonnell ND Weinblatt ME Etanercept therapy in rheumatoid arthritis. A randomized, controlled trial. Ann Intern Med 1999 130 478 86 10075615 10.7326/0003-4819-130-6-199903160-00004

[23] Poulaki V Mitsiades N Mastorakos G Caspi RR Chrousos GP Bouzas E Fas/Fas ligand-associated apoptosis in experimental autoimmune uveoretinitis in rodents: role of proinflammatory corticotropin-releasing hormone. Exp Eye Res 2001 72 623 9 11384150 10.1006/exer.2001.0992

[24] Mitsiades N Poulaki V Mastorakos G Tseleni-Balafouta ST Kotoula V Koutras DA Tsokos M Fas ligand expression in thyroid carcinomas: a potential mechanism of immune evasion. J Clin Endocrinol Metab 1999 84 2924 32 10443700 10.1210/jcem.84.8.5917

[25] Koizumi K Poulaki V Doehmen S Welsandt G Radetzky S Lappas A Kociok N Kirchhof B Joussen AM Contribution of TNF-{alpha} to Leukocyte Adhesion, Vascular Leakage, and Apoptotic Cell Death in Endotoxin-Induced Uveitis In Vivo. Invest Ophthalmol Vis Sci 2003 44 218491 12714660 10.1167/iovs.02-0589

[26] Poulaki V Mitsiades N Romero ME Tsokos M Fas-mediated apoptosis in neuroblastoma requires mitochondrial activation and is inhibited by FLICE inhibitor protein and Bcl-2. Cancer Res 2001 61 4864 72 11406564

[27] Cogan DG Toussaint D Kubawara T Retinal vascular patterns. IV. Diabetic retinopathy. Arch Ophthalmol 1961 66 366 78 13694291 10.1001/archopht.1961.00960010368014

[28] Kern TS Engerman RL Vascular lesions in diabetes are distributed non-uniformly within the retina. Exp Eye Res 1995 60 545 9 7615020 10.1016/s0014-4835(05)80069-7

[29] Mohamed AK Bierhaus A Schiekofer S Tritschler H Ziegler R Nawroth PP The role of oxidative stress and NF-kappaB activation in late diabetic complications. Biofactors 1999 10 157 67 10609877 10.1002/biof.5520100211

[30] Ledebur HC Parks TP Transcriptional regulation of the intercellular adhesion molecule-1 gene by inflammatory cytokines in human endothelial cells. Essential roles of a variant NF-kappa B site and p65 homodimers. J Biol Chem 1995 270 933 43 7822333 10.1074/jbc.270.2.933

[31] Singh AK Gudehithlu KP Patri S Litbarg NO Sethupathi P Arruda JA Dunea G Impaired integration of endothelial progenitor cells in capillaries of diabetic wounds is reversible with vascular endothelial growth factor infusion. Transl Res 2007 149 282 91 17466928 10.1016/j.trsl.2006.11.005

[32] Emanueli C Graiani G Salis MB Gadau S Desortes E Madeddu P Prophylactic gene therapy with human tissue kallikrein ameliorates limb ischemia recovery in type 1 diabetic mice. Diabetes 2004 53 1096 103 15047627 10.2337/diabetes.53.4.1096

[33] Mizutani M Kern TS Lorenzi M Accelerated death of retinal microvascular cells in human and experimental diabetic retinopathy. J Clin Invest 1996 97 2883 90 8675702 10.1172/JCI118746 PMC507384

[34] Barber AJ Lieth E Khin SA Antonetti DA Buchanan AG Gardner TW Neural apoptosis in the retina during experimental and human diabetes. Early onset and effect of insulin. J Clin Invest 1998 102 783 91 9710447 10.1172/JCI2425 PMC508941

[35] Alnemri ES Mammalian cell death proteases: a family of highly conserved aspartate specific cysteine proteases. J Cell Biochem 1997 64 33 42 9015752 10.1002/(sici)1097-4644(199701)64:1<33::aid-jcb6>3.0.co;2-0

[36] Colussi PA Harvey NL Kumar S Prodomain-dependent nuclear localization of the caspase-2 (Nedd2) precursor. A novel function for a caspase prodomain. J Biol Chem 1998 273 24535 42 9733748 10.1074/jbc.273.38.24535

[37] Colussi PA Harvey NL Shearwin-Whyatt LM Kumar S Conversion of procaspase-3 to an autoactivating caspase by fusion to the caspase-2 prodomain. J Biol Chem 1998 273 26566 70 9756894 10.1074/jbc.273.41.26566

[38] Martin PM Roon P Van Ells TK Ganapathy V Smith SB Death of retinal neurons in streptozotocin-induced diabetic mice. Invest Ophthalmol Vis Sci 2004 45 3330 6 15326158 10.1167/iovs.04-0247

[39] Wallach D Varfolomeev EE Malinin NL Goltsev YV Kovalenko AV Boldin MP Tumor necrosis factor receptor and Fas signaling mechanisms. Annu Rev Immunol 1999 17 331 67 10358762 10.1146/annurev.immunol.17.1.331

[40] Walsh K Sata M Is extravasation a Fas-regulated process? Mol Med Today 1999 5 61 7 10200946 10.1016/s1357-4310(98)01415-4

[41] Ohta N Tsai JY Secchi EF Kador PF Sato S Neutrophils in galactose-fed dogs: suppressed apoptosis and increased adhesion to retinal capillary endothelial cells. J Diabetes Complications 1999 13 151 8 10509875 10.1016/s1056-8727(99)00040-9

[42] Luong BT Chong BS Lowder DM Treatment options for rheumatoid arthritis: celecoxib, leflunomide, etanercept, and infliximab. Ann Pharmacother 2000 34 743 60 10860137 10.1345/aph.19344

